# What about proactive language control?

**DOI:** 10.3758/s13423-019-01654-1

**Published:** 2019-08-13

**Authors:** Mathieu Declerck

**Affiliations:** Laboratoire de Psychologie Cognitive, Aix-Marseille Université and Centre National de la Recherche Scientifique, Centre St. Charles, 3 place Victor Hugo, 13331 Marseille, France

**Keywords:** Proactive language control, Reversed language dominance, Language-mixing costs, Blocked language order

## Abstract

While several reviews provide an in-depth discussion on reactive language control, which is the language control process that is initiated when the non-target language disrupts the selection of target language words, few have touched on proactive language control, which is the language control process implemented as an anticipation of any non-target language interference disrupting the selection of target language words. In the current review, three prominent markers of proactive language control are discussed (i.e., the reversed language dominance effect, language-mixing costs, and the blocked language-order effect). Based on these three markers, it appears that proactive language control can be implemented to mainly restrict interference from the first language during bilingual language production, but is typically absent during bilingual language comprehension. The literature also implies that proactive language control might be partly domain general. With respect to the underlying mechanism of proactive language control, there are some indications that proactive language control relies on inhibition, but no unequivocal evidence has been provided so far.

## Introduction

During bilingual language processing, words from the non-target language are activated (e.g., Costa, Caramazza, & Sebastián-Gallés, 2000; Hermans, Bongaerts, De Bot, & Schreuder, 1998; Meade, Midgley, Dijkstra, & Holcomb, 2018) and sometimes even selected by mistake (e.g., Declerck, Lemhöfer, & Grainger, 2017; Gollan, Sandoval, & Salmon, 2011; Poulisse & Bongaerts, 1994). To minimize cross-language interference, and increase the chances that words of the target language are selected, a language control process is implemented. While language control is typically viewed as one process, two types of control processes have been identified in the literature (e.g., Ma, Li, & Guo, 2016; Peeters & Dijkstra, 2018; see also Braver, 2012): reactive language control (also known as transient language control), which is the language control process that is implemented when the non-target language disrupts the selection of target language words, and proactive language control (also known as sustained language control), which is the language control process that is implemented as an anticipation of any non-target language interference disrupting the selection of target language words. Put differently, reactive language control resolves any cross-language interference when it occurs, whereas proactive language control is a preventive control process.

Several reviews have discussed language control (e.g., Bobb & Wodniecka, 2013; Calabria, Costa, Green, & Abutalebi, 2018; Declerck & Philipp, 2015). However, the focus of these reviews is typically on reactive language control, with little attention devoted to proactive language control. This is probably due to most language control studies focusing on reactive language control. Moreover, it is not always easy to identify how bilingual models account for proactive language control. To encourage future research into proactive language control, the current review will focus on this control process by discussing several of its markers.

## Language control models and proactive language control

As noted above, the explanation of proactive language control in most bilingual models is not straightforward, since typically no indication is given about whether the discussed language control process is reactive or proactive. In this section, I will try to deduce how proactive language control is accounted for by bilingual models.

Some bilingual comprehension models seem to mainly rely on proactive language control. The BIA (Grainger & Dijkstra, 1992) and BIA-d (Grainger, Midgley, & Holcomb, 2010), for instance, assume that language control relies on inhibition instigated by language nodes that determine the activation of word representations across each language. More specifically, language control during bilingual language comprehension is a process that is instigated by the stimulus language. This language of the stimulus is assumed to automatically activate its corresponding language node, which should result in inhibition of word representations of the other language. So, it would appear that this language control process is proactive, as the activation of a language automatically and preemptively reduces the activation of words in the other language.

Some bilingual production models also mainly rely on proactive language control. La Heij (2005; see also Poulisse & Bongaerts, 1994), for example, suggested that language representations of the target language receive extra activation from a corresponding language cue at the semantic level. Hence, this additional activation will result in a preemptively higher chance of future target language word(s) being activated.

The models discussed thus far assume that the language control process is in principal proactive, which raises several questions. First, proactive language control is typically viewed as a long-lasting process in the literature, hence its alternative name “sustained language control” (for evidence along these lines, see Christoffels, Ganushchak, & La Heij, 2016). In these models, on the other hand, it is assumed that the proactive language control process possibly changes from word to word, which is more in line with how the literature views reactive language control, also known as “transient language control”. However, it might be more appropriate to interpret the sustained nature of proactive language control as a relative characteristic, with certain situations only requiring proactive language control for a very short time (e.g., a quick demand in English [*“go”*] to a colleague who enters the room while you are on the phone with clients who speak Dutch), whereas other situations require proactive language control over a long time (e.g., when discussing vacation plans with a monolingual friend).

Another issue with these bilingual models is that they do not explicitly account for reactive language control. With respect to this concern, Green and Abutalebi (2013) have proposed some theoretical foundation for the occurrence of both proactive and reactive language control in their bilingual model, and thus by extension several similar bilingual models (Declerck, Koch, & Philipp 2015; Green, 1998; Schwieter & Sunderman, 2008). They hint that goal maintenance, conflict monitoring, and interference suppression could be viewed within the context of proactive and reactive language control. This would entail that proactive language control is triggered by goal maintenance and reactive language control is triggered by conflict monitoring, and both these processes influence bilingual language processing through inhibition.

In sum, while it is not always clear how bilingual models implement proactive language control, we can already see some differences between models that seemingly can account for this process. Some models assume that the main underlying mechanism of proactive language control is inhibition of the non-target language, whereas others assume that additional activation of the target language underlies proactive language control. To investigate this and other issues, some of the prominent markers of proactive language control (i.e., the reversed language dominance effect in mixed language blocks, language-mixing costs, and the blocked language-order effect) are discussed in the following section.

## Markers of proactive language control

### Reversed language dominance in mixed language blocks

The first marker of proactive language control that is discussed is the reversed language dominance effect found in mixed language blocks (i.e., blocks in which two or more languages are processed). This reversed language dominance effect in mixed language blocks is considered reversed because it shows the opposite pattern of what is typically observed in pure language blocks (i.e., blocks in which only one language is processed). More specifically, first language (L1) performance is typically better than second language (L2) performance in pure language blocks (for reviews, see Hanulová, Davidson, & Indefrey, 2011; Runnqvist, Strijkers, Sadat, & Costa, 2011). This effect is assumed to be due to more practice with L1 over a long time, which results in a larger base activation of L1 than L2. In mixed language blocks, on the other hand, several studies have observed worse L1 than L2 performance (e.g., Christoffels et al., 2016; Christoffels, Firk, & Schiller, 2007; Costa & Santesteban, 2004; Heikoop, Declerck, Los, & Koch, 2016; Gollan & Ferreira, 2009; Verhoef, Roelofs, & Chwilla, 2009, 2010). Christoffels et al. (2007), for example, asked Dutch-German bilinguals to name pictures in either Dutch or German based on a cue (red or green color presented with each picture) in mixed language blocks. The results showed that L1 (Dutch) reaction times were slower than L2 (German) reaction times.

#### Explanations

The reversal of the language dominance pattern in mixed language blocks can be explained by assuming that by disadvantaging L1 and/or by favoring L2, more similar levels of L1 and L2 activation will be acquired, and thus overall language processing will be improved in the context of mixed language blocks. More specifically, Costa and Santesteban (2004) proposed that the selection criteria of each language can be altered to achieve this. A more prominent explanation in the literature indicates that L1 is consistently inhibited in mixed language blocks (e.g., Christoffels et al., 2007; Gollan & Feirreira, 2009). Along the same lines, it has been suggested that the reversed language dominance effect could be explained with a constant increase in L2 activation throughout mixed language blocks (Declerck, Thoma, Koch, & Philipp, 2015). So, according to these explanations, the reversed language dominance effect is a measure of proactive language control implemented in mixed language blocks.

#### Exceptions

Prior research has shown that a reversed language dominance effect can occur during bilingual language production in mixed language blocks with an unpredictable (e.g., Costa, Santesteban, & Ivanova, 2006; Li & Gollan, 2018) and predictable language sequence (e.g., Declerck, Stephan, Koch, & Philipp, 2015; Declerck et al., 2013), and during reading aloud of written paragraphs (e.g., Gollan & Goldrick, 2016, 2018; Gollan, Schotter, Gomez, Murillo, & Rayner, 2014; Gollan, Stasenko, Li, & Salmon, 2017), picture naming with sentences (Tarlowski, Wodniecka, & Marzecová, 2013), and picture/digit naming with single words (e.g., Costa & Santesteban, 2004; Heikoop et al., 2016). However, while the reversed language dominance effect can occur in most contexts, the effect does not always occur. As a matter of fact, it is one of the most elusive effects in the language control literature. Next to the reversed language dominance pattern in mixed language blocks, some studies have found no difference between L1 and L2 performance in mixed language blocks (e.g., Calabria, Branzi, Marne, Hernández, & Costa, 2015; Prior & Gollan, 2011), and other still found worse L2 than L1 performance in mixed language blocks (e.g., Ma et al., 2016; Wang, Kuhl, Chen, & Dong, 2009).

While a number of studies investigated the L1-L2 pattern in mixed language blocks, on the surface it is difficult to pinpoint when a reversed language dominance effect would occur. For example, it would be reasonable to assume that the reversed language dominance effect is related to language proficiency, since the difference between L1 and L2 should be larger for second language learners than for highly proficient bilinguals. Consequently, second language learners would benefit more from a relative performance decrease of L1 compared to L2 in mixed language blocks. However, studies comparing second language learners and highly proficient bilinguals found no difference in the L1-L2 pattern across groups, while overall reversed language dominance effects were observed (Costa & Santesteban, 2004; Santesteban & Costa, 2016; see also Schwieter & Sunderman, 2008).

With a large group of Spanish-English bilinguals (cf. Kleinman & Gollan, 2016), Kleinman and Gollan (2018) recently showed that the occurrence of the reversed language dominance effect might be due to the number of trials. In their study, they found that the language dominance pattern reversed more as participants performed more trials, which indicates that it takes time to observe a reversed language dominance effect in a mixed language block. This claim seems to be reflected in the production literature, as studies with a high number of mixed language block trials per participant (> 900 trials) tend to show a reversed language dominance effect (Costa et al., 2006; Costa & Santesteban, 2004; Verhoef et al., 2009, 2010; however, see Fink & Goldrick, 2015; Meuter & Allport, 1999, for studies with over 900 trials per participant that did not show a reversed language dominance effect).

Finally, the literature also indicates that the reversed language dominance effect does not occur in bilingual language comprehension studies, during which L1 and L2 written words have to be categorized in a mixed language block (e.g., Declerck & Grainger, 2017; Hirsch, Declerck, & Koch, 2015; Macizo, Bajo, & Paolieri, 2012; Orfanidou & Sumner, 2005; Thomas & Allport, 2000; Von Studnitz & Green, 1997). Due to the pervasive absence of the reversed language dominance effect during bilingual language comprehension, it might be claimed that no comprehension-based proactive language control is used in mixed language blocks.

#### Methodological issues

When looking into the reversed language dominance effect it becomes obvious that the effect is an extreme case on a continuum, ranging from better L1 than L2 performance to better L2 than L1 performance. So, we are missing out on a lot of information by solely focusing on the reversed language dominance effect. A more accurate and sensitive marker of proactive language control would consist of the comparison between L1 and L2 performance in mixed versus pure language blocks. This measure would give us an idea of how the relative language dominance changed in mixed language blocks compared to the language dominance that is observed in pure language blocks, instead of solely relying on an extreme case of the L1-L2 pattern.

Interestingly, there is another effect in the proactive language control literature that is almost identical to this novel way of calculating the reversed language dominance effect, namely asymmetrical mixing costs (see next section for a full explanation of mixing costs and their asymmetry across languages). The only difference is that the novel reversed language dominance effect contains both switch and repetition trials in mixed language blocks, whereas asymmetrical mixing costs only relies on repetition trials in mixed language blocks. Yet, since the reversed language dominance effect should also occur for repetition trials (e.g., Zheng, Roelofs, & Lemhöfer, 2018), this difference should not really matter. This challenges whether the novel, and possibly the classic, reversed language dominance effect and asymmetrical mixing costs are different markers. If they are the same marker, there is the question of which explanation is correct, since they both rely on different explanations.

### Language-mixing costs

Language-mixing costs, another marker of proactive language control, reflects the performance decrease in trials that are processed in the same language as the previous trial in mixed language blocks (i.e., language-repetition trials) relative to performance in trials in pure language blocks (e.g., Christoffels et al., 2007; Declerck et al., 2013; Ma et al., 2016; Peeters & Dijkstra, 2018; Stasenko, Matt, & Gollan, 2017; Wang et al., 2009; Weissberger, Wierenga, Bondi, & Gollan, 2012). Ma et al. (2016), for example, asked Chinese-English bilinguals to name digits (0–9) in Chinese or English based on a language cue (red or blue circles) in mixed and pure language blocks. The pure language blocks (counterbalanced order of Chinese and English blocks) were presented prior to the mixed language blocks. The results showed that performance during language-repetition trials in mixed language blocks was worse than during trials in pure language blocks. As in most studies (e.g., Christoffels et al., 2007; Jylkkä, Lehtonen, Lindholm, Kuusakoski, & Laine, 2018; Mosca & de Bot, 2017; Peeters & Dijkstra, 2018; Prior & Gollan, 2011; however, see Declerck et al., 2013; Wang et al., 2009), these language-mixing costs were asymmetrical across languages, with larger L1 than L2 mixing costs.^1^

It should be noted that putting the pure language blocks consistently in front of the mixed language blocks, as in the study by Ma et al. (2016), is not the only method to derive language-mixing costs. Others have completely counterbalanced the order of all block types (i.e., L1 pure language, L2 pure language, and mixed language blocks) across participants (e.g., Gollan & Ferreira, 2009). Still others present the pure language blocks (both L1 and L2) consecutively and counterbalance their order relative to the mixed language blocks (e.g., Declerck, Koch, Duñabeitia, Grainger, & Stephan, 2019). Finally, a “sandwich method” can be used with pure language blocks before and after the mixed language blocks, which are sandwiched in the middle. With the latter method, there is even a difference as to whether pure language blocks of both languages are presented before and after the mixed language blocks (e.g., Declerck et al., 2013) or pure language blocks of one language are presented before the mixed language blocks and pure language blocks of the other language are presented after the mixed language blocks (e.g., Stasenko, et al., 2017).

#### Explanations

According to Ma et al. (2016), language-mixing costs occur because in pure language blocks the non-target language is proactively inhibited in order to restrict interference from the non-target language. Additionally, the target language is proactively activated. In the mixed language blocks, little to no proactive inhibition would occur, whereas both languages would be proactively activated. Consequently, more cross-language interference should occur in mixed language blocks than in pure language blocks, and thus performance should be worse in repetition trials of mixed language blocks than in trials of pure language blocks. So, this explanation indicates that language-mixing costs show the difference in proactive language control between mixed and pure language blocks.

It has been proposed, however, that mixing costs are due to more than just proactive language control (e.g., Prior & MacWhinney, 2010). One prominent alternative account is that mixing costs are due to monitoring processes (e.g., Braver, Reynolds, & Donaldson, 2003; Koch, Prinz, & Allport, 2005; Prior & MacWhinney, 2010). Because of the additional attentional demands imposed by the language monitoring process in mixed language blocks, language processing should be worse during language-repetition trials in mixed language blocks than during trials in pure language blocks.

Jylkkä et al. (2018) pointed out that asymmetrical mixing costs are difficult to explain with this account, since one would expect similar monitoring of L1 and L2 in mixed language blocks. Yet, Jylkkä and colleagues did not take into account that more monitoring might be necessary in pure L2 blocks than in pure L1 blocks due to more cross-language interference in the former. In turn, asymmetrical mixing costs could be explained with language monitoring.

In fact, it is very difficult to distinguish between these two accounts of mixing costs, since they are intertwined. According to the proactive language control account, mixing costs occur because of an increase in cross-language interference. In turn, it is generally assumed that an increase in cross-language interference results in an increase in monitoring (e.g., Botvinick, Braver, Barch, Carter, & Cohen, 2001; Declerck, Lemhöfer, et al., 2017). Hence, it might very well be that mixing costs are a measure of more than just proactive language control.

#### Exceptions

Whereas many studies have provided unambiguous evidence for language-mixing costs (e.g., Declerck et al., 2013; Ma et al., 2016; Peeters & Dijkstra, 2018), it should be noted that these studies typically rely on mixed language blocks that require involuntary language switching (i.e., cued and predictable language switching). There are also several contexts in which language-mixing costs are not reliably observed, such as when bilinguals can switch languages on a voluntary basis in the mixed language blocks (de Bruin, Samuel, & Duñabeitia, 2018; Gambi & Hartsuiker, 2016; Gollan & Ferreira, 2009; Gross & Kaushanskaya, 2015; for a discussion, see Blanco-Elorrieta & Pylkkänen, 2018). Gollan and Ferreira (2009), for example, investigated Spanish-English bilinguals while the bilinguals could freely choose the language on each trial in the mixed language blocks (i.e., voluntary language switching). Their results showed L1 mixing costs but an L2 mixing advantage for second language learners. The authors explained this pattern by proposing that second language learners named simple words in L2 in mixed language blocks, whereas more difficult words would be named in L1. However, it should be noted that some studies investigating involuntary language switching also observed an L2 mixing benefit or absent L2 mixing costs next to L1 mixing costs (Christoffels et al., 2007; Jylkkä et al., 2018; Mosca & Clahsen, 2016; Mosca & de Bot, 2017). These findings could not be explained with the same strategy proposed by Gollan and Ferreira (2009). According to Mosca and Clahsen (2016), this pattern could be due to additional resources for the weaker language to make overall processing better, which is similar to the explanations of the reversed language dominance effect.

There is also only minimal evidence for language-mixing costs in the bilingual language comprehension literature. Grainger and Beauvillain (1987) were the first to investigate language-mixing costs during bilingual language comprehension. In their study, French-English bilinguals performed a generalized lexical decision task (i.e., is the presented string of letters a word in either language or not) in mixed and pure language blocks. The results showed no evidence for language-mixing costs. More recently, Declerck et al. (2019) also investigated language-mixing costs during bilingual comprehension across three experiments, examining different bilinguals (French-English and French-Spanish) and different tasks (parity, magnitude, and animacy tasks). Whereas two experiments with French-English bilinguals showed no such cost, language-mixing costs were observed in an experiment with French-Spanish bilinguals. This discrepancy was explained by an increase in parallel language activation with the French-Spanish bilinguals, since the French-Spanish number words contained more cognates than the French-English number and non-numeric words. Hence, a more pronounced proactive language control process would be implemented by the French-Spanish bilinguals.

#### Methodological issues

As indicated at the beginning of the mixing-cost section, only language-repetition trials of mixed language blocks are compared to trials in pure language blocks to compute language-mixing costs. The reason why not all mixed language block trials (i.e., language-repetition and language-switch trials) are compared to trials in pure language blocks is that researchers typically want to keep language-mixing costs disentangled from language-switch costs, which is a measure of reactive language control and consists of worse performance when the previous trial is in a different language (language-switch trials) than when it is in the same language (language-repetition trials) in a mixed language block (e.g., Costa & Santesteban, 2004; Fink & Goldrick, 2015; Meuter & Allport, 1999). By not including the language-switch trials in the mixing-cost analysis, there is no (or at least less) involvement of reactive language control when measuring language-mixing costs.

However, language-mixing costs could still, in part, be due to language-switch trials, and thus partially the result of reactive language control (for a similar idea, see Jylkkä et al., 2018). We know that switching languages is costly relative to repeating the same language across trials in mixed language blocks (e.g., Costa & Santesteban, 2004; Meuter & Allport, 1999; for a review, see Declerck & Philipp, 2015). In turn, it has been found that performance of consecutive trials, and even beyond immediately consecutive trials, is positively correlated in language tasks (e.g., Baayen & Milin, 2010; Taylor & Lupker, 2001). Consequently, it could be that language-switch trials in mixed language blocks negatively affect the language-repetition trials. In turn, overall performance on language-repetition trials in mixed language blocks is worse than on trials in pure language blocks, where no language-switch trials occur. However, there is evidence against this claim from studies that found different patterns with language-mixing costs and language-switch costs (see section The relationship between proactive and reactive language control). Still, it should be taken into account that, to some extent, language-mixing costs might depend on language-switch trials.

The effect of language-switch trials on language-mixing costs could be minimized in several ways. One possibility is to take the reaction time of the previous trial into the mixed effects analysis as a covariate (cf. Baayen & Milin, 2010). This would allow us to take the impact of the size of the previous trial into account. Another method would be to solely use language-repetition trials in mixed language blocks that are preceded by language-repetition trials, since the main influence typically comes from the prior trial (Baayen & Milin, 2010).

### Blocked language order

The third and last marker of proactive language control that is discussed in this review is the blocked language-order effect (e.g., Branzi et al., 2014; Van Assche et al., 2013). This effect reflects worse performance in a pure language block with language X when previously a pure language block in language Y was performed, relative to when the pure language block in language X was performed without a prior pure language block in language Y. Van Assche et al. (2013), for example, asked Dutch-English and Chinese-English bilinguals to perform a verbal fluency task (e.g., produce as many words as possible in 1 min that start with the letter “s”) in two consecutive pure language blocks. A different language had to be used in both these pure language blocks. The results showed that substantially less words were produced in the second than in the first pure language block.

Another way to calculate the blocked language-order effect within subjects, instead of between subjects, is to use three consecutive pure language blocks, with the same language used in pure language blocks 1 and 3 and the other language used in pure language block 2 (Branzi et al., 2014). One could see the effect of blocked language order by comparing the performance in blocks 1 and 3. Throughout the rest of this review, I will use the standard between-subject methodology when discussing the blocked language-order effect.

#### Explanations

It is assumed that during processing of language Y, in the first block, language X is consistently inhibited. This proactive inhibitory process will continue into the next block, and thus would inhibit the target language (i.e., language X) of this second pure language block. Consequently, an overall worse performance of language X should occur in the second block (e.g., Guo et al., 2011; Misra et al., 2012; Van Assche et al., 2013). This explanation indicates that the blocked language-order effect is due to proactive language control in pure language blocks.

#### Exceptions

Whereas the blocked language-order effect has been observed in some studies (e.g., Branzi et al., 2014; Van Assche et al., 2013), several studies have observed facilitation, instead of inhibition, in the second block (Branzi et al., 2014; Misra et al., 2012). Examining Catalan-Spanish bilinguals in a picture-naming task, Branzi et al. (2014) observed that different stimuli in the first and second block led to worse L1 naming in the second than in the first block, whereas no such effect was observed for L2. When the same stimuli were used in the first and second blocks, on the other hand, there was a facilitation effect for L2 naming in the second block relative to performance in the first block. L1 was unaffected when the same stimuli were used in the first and second blocks (for a similar pattern, see Misra et al., 2012). This facilitation effect has been explained with stimulus repetition effects (e.g., Misra et al., 2012), which makes it easier to respond the second time a stimulus is presented. In turn, when no effect of language order was observed for L1 in Misra et al. (2012), this was seen as the result of the L1 inhibitory effect negating the stimulus repetition effect.

The literature also indicates that worse performance in the second block is only observed when participants have to produce in their L2 in the first block and in their L1 in the second block (e.g., Branzi et al., 2014; Van Assche et al., 2013). This asymmetry, together with the asymmetrical mixing costs and the reversed language-dominance effect, will be discussed later in the section *What is affected by proactive language control?*.

Finally, so far no blocked language-order effect has been observed during bilingual language comprehension. Declerck et al. (2019) investigated this effect with two data sets. First, Declerck et al. (2019) combined the data of three separate experiments (a dataset based on a parity or magnitude task with French-English bilinguals, a dataset based on a parity or magnitude task with French-Spanish bilinguals, and a dataset based on an animacy task with French-English bilinguals), which resulted in no blocked language-order effect with a group of 120 bilinguals. However, since these experiments were not designed to investigate the blocked language-order effect, a new experiment was designed in which 58 French-English bilinguals were tested. In each of the two blocks, the participants had to perform a different task, both of which contained different words, to reduce any practice effects: an animacy task (i.e., does the word represent an object that is alive or not) or a size task (i.e., does the word represent an object that is larger or smaller than 1 meter). This experiment also showed no blocked language-order effect, providing further evidence that a blocked language-order effect is difficult to observe during bilingual language comprehension.

#### Methodological issues

One of the major drawbacks of the blocked language-order effect is that due to fatigue or boredom, participants might perform worse in the second block, regardless of whether a different language was used in the first block. Consequently, the blocked language-order effect could be attributed to processes other than proactive language control. On the other hand, training effects might occur, which would diminish or even obscure any blocked language-order effect. One statistical way to account for these confounding factors would be to add the trial number as a covariate in the mixed-effects analysis (cf. Baayen & Milin, 2010).

In the following sections, I go into more detail regarding proactive language control during bilingual language production versus comprehension, the underlying mechanism of proactive language control, and what is affected by proactive language control. Finally, the relationship between proactive language control and reactive language control and proactive cognitive control are discussed.

## Proactive language control during bilingual language production versus comprehension

From the literature overview presented above, it appears that proactive language control can be implemented during bilingual language production, as all three measures were observed in bilingual language production studies (e.g., Christoffels et al., 2007; Heikoop et al., 2016; Peeters & Dijkstra, 2018; Stasenko et al., 2017; Van Assche et al., 2013). The evidence for proactive language control during bilingual language comprehension, on the other hand, is almost absent. More specifically, no reversed language dominance effect (e.g., Hirsch et al., 2015; Macizo et al., 2012) or blocked language-order effect (Declerck et al., 2019) has been observed in a comprehension study. With respect to language-mixing costs, so far only one experiment has shown this effect in a comprehension study (Exp. 6 in Declerck et al., 2019), whereas the other language-mixing cost experiments provide no evidence for proactive language control during bilingual language comprehension (Exps. 5 and 7 in Declerck et al., 2019; Grainger & Beauvillain, 1987). Thus, the evidence seems to indicate that, contrary to bilingual language production, there is little to no role for proactive language control during bilingual language comprehension.

## Proactive language control and inhibition

As the explanations of each of the three markers show, most prominent accounts of proactive language control rely on inhibition, with some exceptions regarding additional activation. However, direct evidence for proactive inhibitory control is scarce. Several electrophysiological (ERP) studies have investigated whether there is proactive inhibitory control. For instance, Misra et al. (2012) found a larger N2, which is a negative ERP signature related to inhibition (e.g., Jackson, Swainson, Cunnington, & Jackson, 2001; Van Boxtel, Van der Molen, Jennings, & Brunia, 2001), for the blocked language-order effect in L1 (however, see Branzi et al., 2014). While this finding is in line with the idea that proactive language control depends on inhibitory control, there is still uncertainty about whether the N2 is a marker of inhibition or whether it is a marker of response conflict (e.g., Nieuwenhuis, Yeung, Van den Wildenberg, & Ridderinkhof, 2003; Yeung, Botvinick, & Cohen, 2004). Hence, at the moment, the N2 signature does not provide conclusive evidence that proactive language control relies on inhibition.

There are also other clues that proactive language control relies on inhibition. Several studies have found that inhibition declines with age (e.g., Andrés, Guerrini, Phillips, & Perfect, 2008; Hasher, Stoltzfus, Zacks, & Rypma, 1991). In turn, if inhibition is the main process during proactive language control, then markers of proactive language control should also deteriorate with age. Along these lines, Weissberger et al. (2012) observed a small but significant increase in language-mixing costs when age increased. However, these age effects do not unequivocally point towards proactive language control mainly relying on inhibition. First, Gollan and Ferreira (2009) did not find an age effect on language-mixing costs. Second, not all studies show a decline of general inhibition with age (e.g., Rey-Mermet & Gade, 2018; Verhaeghen, 2011). Thus, age effects also do not provide indisputable evidence for inhibition underlying proactive language control.

Taken together, while there are some hints that inhibition might play a role during proactive language control, the evidence is not clear-cut. Yet, there is not much research into whether proactive language control relies on inhibition. Additional research should provide more conclusive evidence, one way or another.

## What is affected by proactive language control?

### Global versus local proactive language control

One of the main aims in several studies that examined proactive language control was to investigate whether this process affects the entire language (global) or specific word representations (local; Branzi et al., 2014; Christoffels et al., 2016; Van Assche et al., 2013). For example, Branzi et al. (2014; for a similar manipulation see Van Assche et al., 2013), as discussed above, manipulated whether the same or different concepts were used across blocks with the blocked language-order effect. Evidence for a global proactive language control effect could be found when a blocked language-order effect was observed with different concepts in the two subsequent pure language blocks, since the effect could not be due to specific word representations being influenced from the first to the second block. If no such effect was observed with different concepts across blocks, but it was observed with the same concepts, then this would be evidence for local proactive language control. The results of Branzi et al. (2014) showed that performance was worse in the second block for L1 naming when different concepts were used, thus providing evidence that proactive language control can affect an entire language.

A similar logic to the one used by Branzi et al. (2014), regarding the investigation of global versus local proactive language control with different versus same stimuli, was used by Christoffels et al. (2016). However, Christoffels and colleagues investigated the reversed language dominance effect instead of the blocked language-order effect. In this study, Dutch-English bilinguals had to name pictures in L1 and L2 pure language blocks before (pretest) and after (post-test) a mixed language block. Three different types of stimuli were used, namely stimuli that were used in the pretest pure language blocks, posttest pure language blocks, and a mixed language block (set A), stimuli that were only used in the pretest and post-test pure language blocks (set B), and stimuli that were only used in the posttest pure language block (set C). The results showed a standard language dominance pattern (i.e., better L1 than L2 performance) with sets A and B in the pretest, whereas in the post-test a reversed language dominance effect was observed with both sets of stimuli (i.e., better L2 than L1 performance). The reversal of the language dominance pattern from the pretest to the post-test with set B shows that a reversed language dominance effect can be initiated by a mixed language block with different stimuli (i.e., set A). Since proactive language control affected different stimuli than the ones that initiated it, evidence is found that proactive language control can affect an entire language. Additional support for this claim comes from comparing the language dominance in the pretest with set A, which was also used in the mixed language block, against the language dominance effect in the post-test with set C, as a significant difference was again observed in language dominance across the pretest with set A and post-test with set C. However, no reversed language dominance effect was observed with set C in the post-test, merely a reduction of the language dominance effect compared to that in the pretest (103 ms vs. 35 ms difference between L1 and L2 performance, respectively, for the pretest with set A and post-test with set C).

One observation about these studies is that they focused on whether proactive language control is a global *or* a local process. However, it could very well be that proactive language control is a global *and* a local process, operating on two levels, similar to reactive language control (e.g., Declerck, Koch, et al., 2015; Declerck & Philipp, 2017; Green, 1998). In the model of Green (1998), for instance, it is assumed that reactive language control occurs between task schemas, which are mental representations of a goal (e.g., speak English or speak French), and thus would affect an entire language (global control). Additionally, this model assumes that language control also occurs at the lemma level between translation-equivalent representations (local control). It would be interesting to investigate the possibility of global and local proactive language control in future research.

### One versus both languages

One question that might arise when reading this review is whether proactive language control only affects one language or whether it can affect both languages. Most of the evidence seems to indicate that mainly L1 is affected by proactive language control. This is obvious for the reversed language dominance effect (e.g., Gollan & Ferreira, 2009; Heikoop et al., 2016), where L1 performance is worse than L2 performance in mixed language blocks, but also for language-mixing costs (e.g., Ma et al., 2016; Peeters & Dijkstra, 2018), which are generally larger for L1 than for L2, and the language-order effect (e.g., Branzi et al., 2014; Van Assche et al., 2013), which seems to mainly affect L1.

There is, however, also evidence that both languages are affected by proactive language control: Whereas most studies show asymmetrical mixing costs, with larger L1 than L2 mixing costs, significant L2 mixing costs could still be found in some studies (e.g., Ma et al., 2016). Moreover, some studies even show similar L1 and L2 mixing costs (Declerck et al., 2013; Wang et al., 2009). So, based on these language-mixing cost studies, proactive language control can affect both languages, but typically one language is affected to a larger degree than the other. Yet, as indicated above in the section on exceptions for language-mixing costs, some studies have shown absent L2 mixing costs or even an L2 mixing benefit (Christoffels et al., 2007; de Bruin et al., 2018; Gambi & Hartsuiker, 2016; Gollan & Ferreira, 2009; Gross & Kaushanskaya, 2015; Mosca & Clahsen, 2016; Mosca & de Bot, 2017) next to substantial L1 mixing costs. So, it is not entirely clear at this point whether proactive language control also affects L2.

## Relationship between proactive and reactive language control

According to the literature, markers of proactive and reactive language control allow for the investigation of different processes. Of interest for this claim are studies that looked into language-mixing costs, since they also measured language-switch costs, a marker for reactive language control. These studies almost uniformly show that there is little evidence for a large overlap between language-mixing costs and language-switch costs: Stasenko et al. (2017) recently found that there was no overlap between these two types of costs with a correlation analysis (r ranging from .020 to .189). Furthermore, asymmetrical mixing costs typically coincide with symmetrical switch costs (Christoffels et al., 2007; Gollan & Ferreira, 2009; Mosca & Clahsen, 2016; Mosca & de Bot, 2017; Peeters & Dijkstra, 2018; Prior & Gollan, 2011; however, see Jylkkä et al., 2018; Ma et al., 2016), which entails similar L1 and L2 switch costs, and asymmetrical switch costs, which entails larger L1 than L2 switch costs, typically coincide with symmetrical mixing costs (Declerck et al., 2013; Wang et al., 2009). Moreover, Ma et al. (2016) found that manipulating the response-to-cue interval had no overall effect on language-mixing costs, but language-switch costs decreased with an increasing response-to-cue interval. Hence, language-mixing costs and language-switch costs responded differently to this manipulation. A discrepancy between language-mixing costs and language-switch costs could also be found when manipulating the cue-to-stimulus interval (Stasenko et al., 2017; however, see Ma et al., 2016), cognate status (Christoffels et al., 2007), experiment half (Stasenko et al., 2017), daily-life language switching frequency (Prior & Gollan, 2011), and short-term training (Prior & Gollan, 2013). However, Weissberger et al. (2012) did find a similarity: Older bilinguals had larger language-mixing costs and language-switch costs than younger bilinguals. A subsequent analysis where age effects for language-mixing costs and language-switch costs were directly compared showed that language-mixing costs and language-switch costs were similarly affected by age for reaction time. However, there was a trend in the error analysis for a more robust age effect on language-switch costs than on language-mixing costs.

Since almost every study seems to show differences between language-mixing costs and language-switch costs, we could deduce that proactive and reactive language control are two separate processes. However, these differences between language-mixing costs and -switch costs are somewhat surprising, as there are several confounds that would increase the chances of observing a connection between mixing costs and switch costs. First, as suggested in the section on mixing costs, due to carry-over effects across trials from switch to repetition trials in mixed language blocks, the connection between mixing costs and switch costs might be overestimating the relationship between proactive and reactive language control. Another confound is that mixing costs and switch costs both rely on repetition trials (Segal, Stasenko, & Gollan, 2019). This connection means that whenever repetition trials are, for example, slow in a specific condition, switch costs are small in the reaction times and mixing costs are large. These confounding connections suggest that mixing costs and switch costs are not the ideal markers to compare proactive and reactive language control. More distinct markers should be considered in future research to compare proactive and reactive language control.

It should also be noted that even if proactive and reactive language control rely on different underlying mechanisms, this does not necessarily mean that these two language control processes are not interconnected. Because proactive and reactive language control have the same goal (i.e., cross-language interference resolution), they might be connected. For instance, it might be that whenever there is an increase in proactive language control, less reactive language control would be necessary, because the cross-language interference would be resolved for the most part by proactive language control.

## Relationship between proactive language control and proactive cognitive control

Several, but not all (Declerck, Koch, et al., 2015; Grainger et al., 2010), bilingual models assume that language control relies on domain-general control processes (e.g., Green, 1998; Schwieter & Sunderman, 2008). There is evidence to support domain-general reactive language control (e.g., Declerck, Grainger, Koch, & Philipp, 2017; Prior & Gollan, 2011; for a review, see Calabria, Baus, & Costa, 2019). Yet, not all studies found evidence along these lines (e.g., Branzi, Calabria, Boscarino, & Costa, 2016; Calabria et al., 2015; see also Gollan, Kleinman, & Wierenga, 2014). These contradictory findings have led to the interim consensus that reactive language control consists partially of domain-general control processes.

There have also been several studies that provide insight into whether proactive language control is domain general. All of these studies relied on mixing costs to investigate the relationship between proactive language control and proactive cognitive control, as mixing costs provide the most straightforward comparison between language control, through language-mixing costs, and more domain-general cognitive control, through task-mixing costs (however, see Jylkkä et al., 2018).

Task-mixing costs are methodologically similar to language-mixing costs. The main difference is that in the mixed blocks participants are required to switch between two different tasks (e.g., parity and magnitude task), instead of switching between two different languages. The task-repetition trials of these mixed task blocks are then compared to trials of pure task blocks, which typically results in task-mixing costs (e.g., Philipp, Kalinich, Koch, & Schubotz, 2008; Rubin & Meiran, 2005; for a review, see Kiesel et al., 2010).

Similar to the reactive language control literature, the evidence for an overlap between proactive language control and proactive cognitive control is somewhat complicated. On the one hand, several studies observed a similar pattern for language- and task-mixing costs. Stasenko et al. (2017), for example, found that both language- and task-mixing costs increased with smaller cue-to-stimulus intervals. Moreover, they observed significant correlations between language- and task-mixing costs with short and long cue-to-stimulus intervals (r ranging from .379 to .421). Along the same lines, Prior and Gollan (2013) observed a significant correlation between language- and task-mixing costs (r = .45), as did Timmer, Calabria, and Costa (2019) after the bilingual participants had language switching training (r = .273).

Timmer et al. (2019), however, found no significant correlation between the two types of mixing costs in the pre-training phase (r = .198). Stasenko et al. (2017) found additional evidence for a difference, as language-mixing costs increased from the first to the second half of the experiment, whereas task-mixing costs decreased from the first to the second half of the experiment. Finally, Weissberger et al. (2012) found that language-mixing costs were larger for older than for younger bilinguals, whereas there was no difference in task-mixing costs related to age.

Taken together, it appears that proactive language control could rely on domain-general control processes, which is in line with several bilingual models that assume language control to be domain general (e.g., Green, 1998; Schwieter & Sunderman, 2008). However, it should be noted that the results across studies indicate that this is, at best, only partially true, with some language-specific control processes still being implemented.

## Summary

Three markers of proactive language control were scrutinized in this review article to examine proactive language control during both bilingual language production and comprehension. In addition, the nature of this process was discussed (i.e., underlying mechanism and functional locus), as were the relationships between proactive language control and reactive language control and proactive cognitive control.

This review indicates that some findings regarding proactive language control are quite unequivocal. One such example is that proactive language control can be implemented during bilingual language production, whereas little evidence for proactive language control has been observed in the context of bilingual language comprehension. Another clear-cut finding is that proactive language control can affect an entire language, mainly L1.

Equally important is that this review shows that many questions still remain. For example, we know very little about whether proactive language control is also a local process that directly affects specific word representations. Another question that remains concerns the underlying mechanism(s) of proactive language control. While there are some clues that proactive language control relies on inhibition, the evidence is far from straightforward. At this point there is also no unequivocal evidence that proactive and reactive language control rely on different mechanisms. Moreover, the relationship between proactive language control and proactive cognitive control is not clear. The literature, thus far, indicates that proactive language control and proactive cognitive control rely partly on the same mechanisms, but proactive language control seems to be partly language specific. It would be fruitful for our understanding of language control specifically, and for bilingual language processing in general, to further explore these basic questions regarding proactive language control.

## Footnotes

1 While in Experiments 1 and 3 of Ma et al. (2016) this was the case, larger L2 than L1 mixing costs were observed in Experiment 2.

## References

[CR1] Andrés P, Guerrini C, Phillips LH, Perfect TJ (2008). Differential effects of aging on executive and automatic inhibition. Developmental Neuropsychology.

[CR2] Baayen HR, Milin P (2010). Analyzing reaction times. International Journal of Psychological Research.

[CR3] Blanco-Elorrieta, E., & Pylkkänen, L. (2018). Ecological validity in bilingualism research and the bilingual advantage. Trends in Cognitive Sciences, 22, 1117–1126 10.1016/j.tics.2018.10.001 30449317

[CR4] Bobb SC, Wodniecka Z (2013). Language switching in picture naming: What asymmetric switch costs (do not) tell us about inhibition in bilingual speech planning. Journal of Cognitive Psychology.

[CR5] Botvinick MM, Braver TS, Barch DM, Carter CS, Cohen JD (2001). Conflict monitoring and cognitive control. Psychological Review.

[CR6] Branzi FM, Calabria M, Boscarino ML, Costa A (2016). On the overlap between bilingual language control and domain-general executive control. Acta Psychologica.

[CR7] Branzi FM, Martin CD, Abutalebi J, Costa A (2014). The after-effects of bilingual language production. Neuropsychologia.

[CR8] Braver TS (2012). The variable nature of cognitive control: A dual mechanisms framework. Trends in Cognitive Sciences.

[CR9] Braver TS, Reynolds JR, Donaldson DI (2003). Neural mechanisms of transient and sustained cognitive control during task switching. Neuron.

[CR10] Calabria M, Baus C, Costa A, Schwieter JW (2019). Cross-talk between language and executive control. *The handbook of the neuroscience of multilingualism*.

[CR11] Calabria M, Branzi FM, Marne P, Hernández M, Costa A (2015). Age-related effects over bilingual language control and executive control. Bilingualism: Language and Cognition.

[CR12] Calabria M, Costa A, Green DW, Abutalebi J (2018). Neural basis of bilingual language control. Annals of the New York Academy of Sciences.

[CR13] Christoffels IK, Firk C, Schiller NO (2007). Bilingual language control: An event-related brain potential study. Brain Research.

[CR14] Christoffels I, Ganushchak L, La Heij W, Schwieter JW (2016). When L1 suffers: Sustained, global slowing and the reversed language effect in mixed language context. *Cognitive control and consequences of multilingualism*.

[CR15] Costa A, Caramazza A, Sebastián-Gallés N (2000). The cognate facilitation effect: Implications for models of lexical access. Journal of Experimental Psychology: Learning, Memory, and Cognition.

[CR16] Costa A, Santesteban M (2004). Lexical access in bilingual speech production: Evidence from language switching in highly proficient bilinguals and L2 learners. Journal of Memory and Language.

[CR17] Costa A, Santesteban M, Ivanova I (2006). How do highly proficient bilinguals control their lexicalization process? Inhibitory and language-specific selection mechanisms are both functional. Journal of Experimental Psychology: Learning, Memory, and Cognition.

[CR18] de Bruin A, Samuel AG, Duñabeitia JA (2018). Voluntary language switching: When and why do bilinguals switch between their languages?. Journal of Memory and Language.

[CR19] Declerck M, Grainger J (2017). Inducing asymmetrical switch costs in bilingual language comprehension by language practice. Acta Psychologica.

[CR20] Declerck M, Grainger J, Koch I, Philipp AM (2017). Is language control just a form of executive control? Evidence for overlapping processes in language switching and task switching. Journal of Memory and Language.

[CR21] Declerck M, Koch I, Duñabeitia JA, Grainger J, Stephan DN (2019). What absent switch costs and mixing costs during bilingual language comprehension can tell us about language control. Journal of Experimental Psychology: Human Perception and Performance.

[CR22] Declerck M, Koch I, Philipp AM (2015). The minimum requirements of language control: Evidence from sequential predictability effects in language switching. Journal of Experimental Psychology: Learning, Memory, and Cognition.

[CR23] Declerck M, Lemhöfer K, Grainger J (2017). Bilingual language interference initiates error detection: Evidence from language intrusions. Bilingualism: Language and Cognition.

[CR24] Declerck M, Philipp AM (2017). Is there lemma-based language control? The influence of language practice and language-specific item practice on asymmetrical switch costs. Language, Cognition and Neuroscience.

[CR25] Declerck M, Philipp AM (2015). A review of control processes and their locus in Language switching. Psychonomic Bulletin & Review.

[CR26] Declerck M, Philipp AM, Koch I (2013). Bilingual control: Sequential memory in language switching. Journal of Experimental Psychology: Learning, Memory, and Cognition.

[CR27] Declerck M, Stephan DN, Koch I, Philipp AM (2015). The other modality: Auditory stimuli in language switching. Journal of Cognitive Psychology.

[CR28] Declerck M, Thoma AM, Koch I, Philipp AM (2015). Highly proficient bilinguals implement inhibition - Evidence from n-2 language repetition costs when switching between three languages. Journal of Experimental Psychology: Learning, Memory, and Cognition.

[CR29] Fink A, Goldrick M (2015). Pervasive benefits of preparation in language switching. Psychonomic Bulletin & Review.

[CR30] Gambi C, Hartsuiker RJ (2016). If you stay, it might be easier: Switch costs from comprehension to production in a joint switching task. Journal of Experimental Psychology: Learning, Memory, and Cognition.

[CR31] Gollan TH, Ferreira VS (2009). Should I stay or should I switch? A cost-benefit analysis of voluntary language switching in young and aging bilinguals. Journal of Experimental Psychology: Learning, Memory, and Cognition.

[CR32] Gollan TH, Goldrick M (2016). Grammatical constraints on language switching: Language control is not just executive control. Journal of Memory and Language.

[CR33] Gollan TH, Goldrick M (2018). A switch is not a switch: Syntactically-driven bilingual language control. Journal of Experimental Psychology: Learning, Memory, and Cognition.

[CR34] Gollan TH, Kleinman D, Wierenga CE (2014). What’s easier: Doing what you want, or being told what to do? Cued versus voluntary language and task switching. Journal of Experimental Psychology: General.

[CR35] Gollan TH, Sandoval T, Salmon DP (2011). Cross-language intrusion errors in aging bilinguals reveal the link between executive control and language selection. Psychological Science.

[CR36] Gollan TH, Schotter ER, Gomez J, Murillo M, Rayner K (2014). Multiple levels of bilingual language control: Evidence from language intrusions in reading aloud. Psychological Science.

[CR37] Gollan TH, Stasenko A, Li C, Salmon DP (2017). Bilingual language intrusions and other speech errors in Alzheimer’s disease. Brain and Cognition.

[CR38] Grainger J, Beauvillain C (1987). Language blocking and lexical access in bilinguals. Quarterly Journal of Experimental Psychology.

[CR39] Grainger J, Dijkstra T, Harris RJ (1992). On the representation and use of language information in bilinguals. *Cognitive processing in bilinguals*.

[CR40] Grainger J, Midgley KJ, Holcomb PJ, Kail M, Hickman M (2010). Re-thinking the bilingual interactive- activation model from a developmental perspective (BIA-d). *Language acquisition across linguistic and cognitive system*s.

[CR41] Green DW (1998). Mental control of the bilingual lexico-semantic system. Bilingualism: Language and Cognition.

[CR42] Green DW, Abutalebi J (2013). Language control in bilinguals: The adaptive control hypothesis. Journal of Cognitive Psychology.

[CR43] Gross M, Kaushanskaya M (2015). Voluntary language switching in English–Spanish bilingual children. Journal of Cognitive Psychology.

[CR44] Guo T, Liu H, Misra M, Kroll JF (2011). Local and global inhibition in bilingual word production: fMRI evidence from Chinese–English bilinguals. NeuroImage.

[CR45] Hanulová J, Davidson DJ, Indefrey P (2011). Where does the delay in L2 picture naming come from? Psycholinguistic and neurocognitive evidence on second language word production. Language and Cognitive Processes.

[CR46] Hasher L, Stoltzfus ER, Zacks RT, Rypma B (1991). Age and inhibition. Journal of Experimental Psychology: Learning, Memory, and Cognition.

[CR47] Heikoop KW, Declerck M, Los SA, Koch I (2016). Dissociating language-switch costs from cue-switch costs in bilingual language switching. Bilingualism: Language and Cognition.

[CR48] Hermans D, Bongaerts T, De Bot K, Schreuder R (1998). Producing words in a foreign language: Can speakers prevent interference from their first language?. Bilingualism: Language and Cognition.

[CR49] Hirsch P, Declerck M, Koch I (2015). Exploring the functional locus of language switching: Evidence from a PRP paradigm. Acta Psychologica.

[CR50] Jackson GM, Swainson R, Cunnington R, Jackson SR (2001). ERP correlates of executive control during repeated language switching. Bilingualism: Language and Cognition.

[CR51] Jylkkä J, Lehtonen M, Lindholm F, Kuusakoski A, Laine M (2018). The relationship between general executive functions and bilingual switching and monitoring in language production. Bilingualism: Language and Cognition.

[CR52] Kiesel A, Steinhauser M, Wendt M, Falkenstein M, Jost K, Philipp AM, Koch I (2010). Control and interference in task switching—A review. Psychological Bulletin.

[CR53] Kleinman D, Gollan TH (2016). Speaking two languages for the price of one: Bypassing language control mechanisms via accessibility-driven switches. Psychological Science.

[CR54] Kleinman D, Gollan TH (2018). Inhibition accumulates over time at multiple processing levels in bilingual language control. Cognition.

[CR55] Koch I, Prinz W, Allport A (2005). Involuntary retrieval in alphabet-arithmetic tasks: Task-mixing and task-switching costs. Psychological Research.

[CR56] La Heij W, Kroll JF, de Groot AMB (2005). Selection processes in monolingual and bilingual lexical access. *Handbook of bilingualism: Psycholinguistic approaches*.

[CR57] Li, C., & Gollan, T. H. (2018). Cognates facilitate switches and then confusion: Contrasting effects of cascade versus feedback on language selection. Journal of Experimental Psychology: Learning, Memory, and Cognition, 44, 974–991 10.1037/xlm0000497 PMC7229571 29283605

[CR58] Ma F, Li S, Guo T (2016). Reactive and proactive control in bilingual word production: An investigation of influential factors. Journal of Memory and Language.

[CR59] Macizo P, Bajo T, Paolieri D (2012). Language switching and language competition. Second Language Research.

[CR60] Meade G, Midgley KJ, Dijkstra T, Holcomb PJ (2018). Cross-language neighborhood effects in learners indicative of an integrated lexicon. Journal of Cognitive Neuroscience.

[CR61] Meuter RFI, Allport A (1999). Bilingual language switching in naming: Asymmetrical costs of language selection. Journal of Memory and Language.

[CR62] Misra M, Guo T, Bobb SC, Kroll JF (2012). When bilinguals choose a single word to speak: Electrophysiological evidence for inhibition of the native language. Journal of Memory and Language.

[CR63] Mosca M, Clahsen H (2016). Examining language switching in bilinguals: The role of preparation time. Bilingualism: Language and Cognition.

[CR64] Mosca M, de Bot K (2017). Bilingual language switching: Production vs. Recognition. Frontiers in Psychology.

[CR65] Nieuwenhuis S, Yeung N, Van Den Wildenberg W, Ridderinkhof KR (2003). Electrophysiological correlates of anterior cingulate function in a go/no-go task: Effects of response conflict and trial type frequency. Cognitive, Affective, & Behavioral Neuroscience.

[CR66] Orfanidou E, Sumner P (2005). Language switching and the effects of orthographic specificity and response repetition. Memory & Cognition.

[CR67] Peeters D, Dijkstra T (2018). Sustained inhibition of the native language in bilingual language production: A virtual reality approach. Bilingualism: Language and Cognition.

[CR68] Philipp AM, Kalinich C, Koch I, Schubotz RI (2008). Mixing costs and switch costs when switching stimulus dimensions in serial predictions. Psychological Research.

[CR69] Poulisse N, Bongaerts T (1994). First language use in second language production. Applied Linguistics.

[CR70] Prior A, Gollan TH (2011). Good language-switchers are good task-switchers: Evidence from Spanish-English and Mandarin-English bilinguals. Journal of the International Neuropsychological Society.

[CR71] Prior A, Gollan TH (2013). The elusive link between language control and executive control: A case of limited transfer. Journal of Cognitive Psychology.

[CR72] Prior A, MacWhinney B (2010). A bilingual advantage in task switching. Bilingualism: Language and Cognition.

[CR73] Rey-Mermet A, Gade M (2018). Inhibition in aging: What is preserved? What declines? A meta-analysis. Psychonomic Bulletin & Review.

[CR74] Rubin O, Meiran N (2005). On the origins of the task mixing cost in the cuing task-switching paradigm. Journal of Experimental Psychology: Learning, Memory, and Cognition.

[CR75] Runnqvist E, Strijkers K, Sadat J, Costa A (2011). On the temporal and functional origin of L2 disadvantages in speech production: A critical review. Frontiers in Psychology.

[CR76] Santesteban M, Costa A, Schwieter JW (2016). Are cognate words “special”? On the role of cognate words in language switching performance. *Cognitive control and consequences of multilingualism*.

[CR77] Schwieter JW, Sunderman G (2008). Language switching in bilingual speech production: In search of the language-specific selection mechanism. The Mental Lexicon.

[CR78] Segal D, Stasenko A, Gollan TH (2019). More evidence that a switch is not (always) a switch: Binning bilinguals reveals dissociations between task and language switching. Journal of Experimental Psychology: General.

[CR79] Stasenko A, Matt GE, Gollan TH (2017). A relative bilingual advantage in switching with preparation: Nuanced explorations of the proposed association between bilingualism and task switching. Journal of Experimental Psychology: General.

[CR80] Tarlowski A, Wodniecka Z, Marzecová A (2013). Language switching in the production of phrases. Journal of Psycholinguistic Research.

[CR81] Taylor TE, Lupker SJ (2001). Sequential effects in naming: A time-criterion account. Journal of Experimental Psychology: Learning, Memory, and Cognition.

[CR82] Thomas MSC, Allport A (2000). Language switching costs in bilingual visual word recognition. Journal of Memory and Language.

[CR83] Timmer K, Calabria M, Costa A (2019). Non-linguistic effects of language switching training. Cognition.

[CR84] Van Assche E, Duyck W, Gollan TH (2013). Whole-language and item-specific control in bilingual language production. Journal of Experimental Psychology: Learning, Memory, and Cognition.

[CR85] Van Boxtel GJ, van der Molen MW, Jennings JR, Brunia CH (2001). A psychophysiological analysis of inhibitory motor control in the stop-signal paradigm. Biological Psychology.

[CR86] Verhaeghen P (2011). Aging and executive control: Reports of a demise greatly exaggerated. Current Directions in Psychological Science.

[CR87] Verhoef KMW, Roelofs A, Chwilla DJ (2009). Role of inhibition in language switching: Evidence from event-related brain potentials in overt picture naming. Cognition.

[CR88] Verhoef KMW, Roelofs A, Chwilla DJ (2010). Electrophysiological evidence for endogenous control of attention in switching between languages in overt picture naming. Journal of Cognitive Neuroscience.

[CR89] Von Studnitz RE, Green DW (1997). Lexical decision and language switching. International Journal of Bilingualism.

[CR90] Wang Y, Kuhl PK, Chen C, Dong Q (2009). Sustained and transient language control in the bilingual brain. NeuroImage.

[CR91] Weissberger GH, Wierenga CE, Bondi MW, Gollan TH (2012). Partially over- lapping mechanisms of language and task control in young and older bilinguals. Psychology and Aging.

[CR92] Yeung N, Botvinick MM, Cohen JD (2004). The neural basis of error detection: Conflict monitoring and the error-related negativity. Psychological Review.

[CR93] Zheng X, Roelofs A, Lemhöfer K (2018). Language selection errors in switching: Language priming or cognitive control?. Language, Cognition and Neuroscience.

